# Deformation Monitoring of Earth Fissure Hazards Using Terrestrial Laser Scanning

**DOI:** 10.3390/s19061463

**Published:** 2019-03-26

**Authors:** Yunfeng Ge, Huiming Tang, Xulong Gong, Binbin Zhao, Yi Lu, Yong Chen, Zishan Lin, Hongzhi Chen, Yashi Qiu

**Affiliations:** 1 Faculty of Engineering, China University of Geosciences, Wuhan 430074, China; 13972521903@cug.edu.cn (Y.C.); zishanlin@cug.edu.cn (Z.L.); 20131002930@cug.edu.cn (H.C.); qiuyashi@cug.edu.cn (Y.Q.); 2 Key Laboratory of Earth Fissures Geological Disaster, Ministry of Land and Resources, Geological Survey of Jiangsu Province, Nanjing 210049, China; xulonggong@126.com (X.G.); lynju@163.com (Y.L.); 3 Three Gorges Research Center for Geo-hazard, Ministry of Education, China University of Geosciences, Wuhan 430074, China; 4 Research Institute of Transmission and Transformation Projects, China Electric Power Research Institute Co., Ltd., State Grid Corporation of China, Beijing 100192, China; zhaobinbin@epri.sgcc.com.cn

**Keywords:** earth fissure hazards, deformation monitoring, terrestrial laser scanning, local displacement, global displacement

## Abstract

Deformation monitoring is a powerful tool to understand the formation mechanism of earth fissure hazards, enabling the engineering and planning efforts to be more effective. To assess the evolution characteristics of the Yangshuli earth fissure hazard more completely, terrestrial laser scanning (TLS), a remote sensing technique which is regarded as one of the most promising surveying technologies in geohazard monitoring, was employed to detect the changes to ground surfaces and buildings in small- and large-scales, respectively. Time-series of high-density point clouds were collected through 5 sequential scans from 2014 to 2017 and then pre-processing was performed to filter the noise data of point clouds. A tiny deformation was observed on both the scarp and the walls, based on the local displacement analysis. The relative height differences between the two sides of the scarp increase slowly from 0.169 m to 0.178 m, while no obvious inclining (the maximum tilt reaches just to 0.0023) happens on the two walls, based on tilt measurement. Meanwhile, global displacement analysis indicates that the overall settlement slowly increases for the ground surface, but the regions in the left side of scarp are characterized by a relatively larger vertical displacement than the right. Furthermore, the comparisons of monitoring results on the same measuring line are discussed in this study and TLS monitoring results have an acceptable consistency with the global positioning system (GPS) measurements. The case study shows that the TLS technique can provide an adequate solution in deformation monitoring of earth fissure hazards, with high effectiveness and applicability.

## 1. Introduction

An earth fissure, also called a ground fissure, ground crack, or surface rapture, is a common natural hazard, posing a heavy threat to human life and infrastructure construction. The category of earth fissures varies according to the following induced factors: (1) Tectonic activities, such as earthquakes, active faults, and landslides [1]; (2) intensive underground excavation, for example, groundwater withdrawal, underground resource mining, and underground engineering; and (3) multiple triggers. To better understand the formation mechanism and evolution process, it becomes extremely necessary to perform deformation monitoring of earth fissure hazards. Therefore, field monitoring remains a critical concern in risk management for earth fissure deformation issues, serving for potential hazard detection and failure prediction. To date, several techniques are available to perform deformation monitoring of earth fissure hazards and land subsidence. The global navigation satellite system (GNSS) technology has been recommended as a powerful tool to map the land subsidence. Sato (2003) selected three baselines to measure the height difference of clay layers in Japan, using global positioning system (GPS) [2]. Abidin (2008) estimated the subsidence rates of four types of land subsidence in Indonesia, based on GPS and leveling surveys [3]. The subsidence phenomenon and vertical movement in the Po Plain Basin was investigated based on more than 100 GPS observation stations [4]. Ustun (2010) detected the ground vertical displacement caused by groundwater withdrawal in the Konya Closed Basin through GNSS [5]. Although these engineering applications have revealed the high accuracy and simple operation of GNSS, it is a point-based method which only can provide the displacement of a single point. Worse, a continuously-operating GPS survey is always time-consuming and vulnerable to external interference. The deformation information, with great extensions, seems to be possible by employing remote sensing technology, such as Synthetic Aperture Radar Interferometry (InSAR) and Terrestrial Laser Scanning (TLS) [6,7,8,9,10]. Among these, TLS, a ground-based remote sensing technique, is a rapid method to acquire surface topography with a high resolution and accuracy of surrounding objects, in the manner of a 3D point cloud (XYZ coordinates), which has been widely used in a range of rock mass instability monitoring. TLS technology was regarded as an effective solution to provide accurate kinematic information of inaccessible regions in landslide and slope hazards and the 3D displacements can be obtained based on the iterative closest points (ICP) algorithm [11,12], which is commonly used to find the minimum difference between two sequential point clouds through iteratively revising the transformation matrix [13]. Some algorithms similar to ICP were employed to monitor landslides, including the shortest distance comparison (SDC) [14] and digital elevation models (DEMs) subtractions [15]. Meanwhile, TLS also has been applied in monitoring other types of natural hazards and engineering practices. Abellán (2009) applied the nearest neighbour averaging (NNA) algorithm to enhance the measurement resolution to millimetric magnitude when mapping a rockfall in Spain, based on a TLS survey [16]. Rosser (2005) insisted that monitoring on a coastal cliff face was necessary to reveal the mechanisms of erosion evolution and employed TLS to collect the detailed cliff process monitoring data for 16 months [17]. The quality evaluation of support in tunnel excavation can be conducted by calculating the surface deformation pre- and post-support installation, based on the point clouds [18]. In addition, Zhou (2014) and Tong (2015) performed mapping and displacement monitoring in underground mining and open-pit areas, respectively, to prove the feasibility of this technology [19,20]. However, the application for earth fissures still requires development. The advantages of TLS, shown in engineering practice, make it possible to collect spatial deformation data of earth fissure hazards remotely and accurately with a simple tripod, without high installation expenses. However, an earth fissure hazard is not completely equivalent to a landslide or rockfall. The region of interest (ROI) has always been placed on the ground surface for the earth fissure, rather than the slope surface. Additionally, distinctive deformation features presented in different types of geological hazards will result in different data sampling and processing.

To determine the feasibility and reliability of TLS on deformation monitoring, the earth fissure hazard in the city of Wuxi was selected for the case study. Repeated TLS surveys were undertaken in 4 years, from 2014 to 2017, to acquire the sets of point clouds. Both local and global displacement were investigated after the filtering work, based on the time series of point clouds, providing an overview of the evolution process of the hazard. We compare the TLS and GPS monitoring results to validate the reliability of the TLS technique, indicating that the two methods have an acceptable consistency.

## 2. Study Area

**Figure 1:**
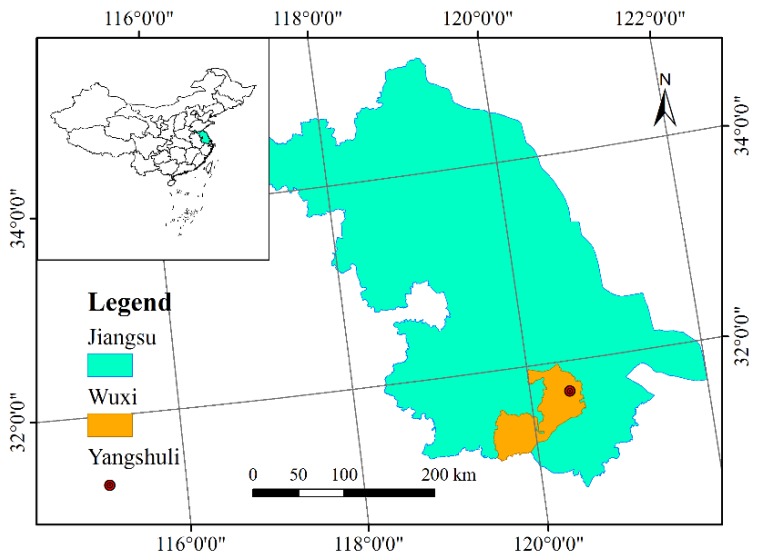
The study area location.

**Figure 2:**
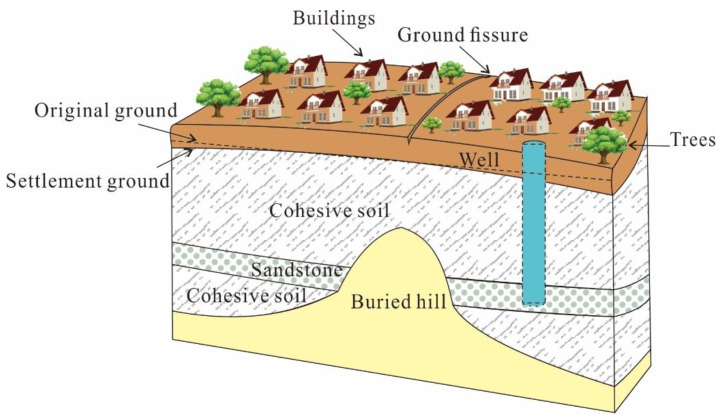
The main formation mechanics of the earth fissure in Wuxi.

The earth fissure hazard studied in this article is located in Yangshuli Village, Wuxi city, Jiangsu Province, in the southeast of China, with geographical coordinates of 31°42′24.68′′ N, 120°26′52.30′′ E (Figure 1). In Jiangsu Province, the earth fissure first appeared in the early 1970s. With the rapid process of urbanization, the demand for underground energy and the development of underground space are increasingly augmented. Correspondingly, more and more earth fissures were discovered in recent years, especially in the region of Suzhou, Wuxi, and Changzhou (SXC). There are many factors contributing to the formation and intensity of earth fissure hazards in this study area and special geological conditions and extensive groundwater withdrawal are the main factors [1]. The basic pattern of geological conditions in the SXC region is mainly formed by Indosinian movement and Yanshanian movement. The Indosinian movement forced the old strata of the Lower and Middle Triassic to suffer intense compression, forming a series of fold systems arranged in a close NNE direction. The Yanshanian movement is mainly characterized by faulting and accompanied by intense magmatic activity. Recent geological tectonic activities mainly show inherited differential movement of uplift and subsidence based on the faults, leading to the great changes in the sedimentary environment from terrestrial hills to coastal areas. The fold systems become the basement, with undulating surfaces overlaid by sediment. Anticline covered by quaternary sedimentary layers is the so-called buried hill [21,22]. The presence of the buried hill greatly impacts the geological and hydro-geological structure models. The thickness of overlying varies on two sides of the buried hill ridge, leading to the difference in consolidation and stress of the soil materials. Worse, the over-exploitation exacerbated this situation and made the fissures generate on the ground surface (Figure 2).

## 3. Data Preparation

### 3.1. Laser Scan Testing

**Figure 3:**
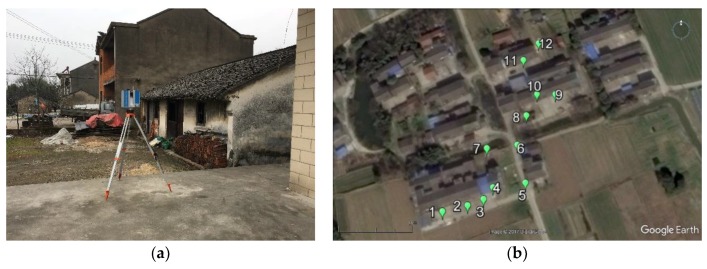

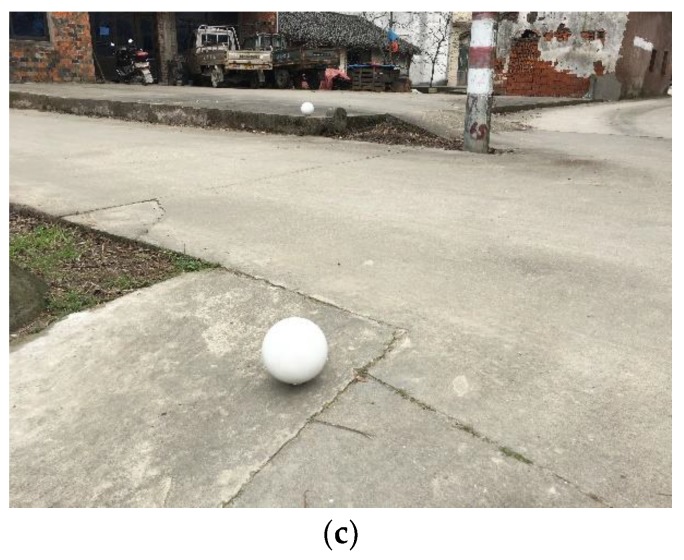
(**a**) Z+F IMAGER 5010C laser scanner; (**b**) the plan view of the study area and the locations of 12 measurement stations; (**c**) white balls used for merging point clouds.

The terrestrial laser scanner Z+F IMAGER 5010C (Zoller + Fröhlich GmbH, Wangen im Allgäu, Germany) was employed to monitor the displacement of the ground surface and buildings. The IMAGER 5010C is a phase-based laser scanner and has the capacity to acquire high-quality data over an appropriate distance (Figure 3a). In total, five scans have been performed to collect the time-series of five point clouds between 2014 and 2017. Prior to each measurement, several scanning parameters, such as scanning distance, resolution, and quality, need to be chosen carefully. The resolution and scanning distance jointly control the point interval and point density and the quality is mainly related to the noise reduction. For acquiring accurate geometrical information of targets, both the resolution and quality were set as high level and the scanning distance was assigned to be less than 50 m. Twelve measurement stations, with a distance of about 20 m, were installed to capture local data for each scanning due to the large-scale region, and the entire study area can be represented by merging 12 sets of point clouds into one (Figure 3b). The scanning distance between the scanner and the target varies with each station, therefore it is impossible to obtain a constant resolution (point interval) for the whole set of TLS data. However, the average resolution can be determined through calculating the mean value of point intervals at different scanning distances (Table 1). Several white balls were placed on the ground surface as reference objects for the convenience of point cloud registration and merging (Figure 3c).

### 3.2. Data Pre-Processing

**Figure 4:**
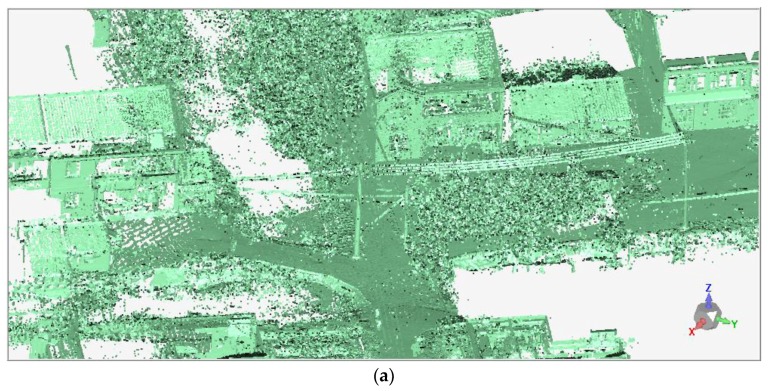

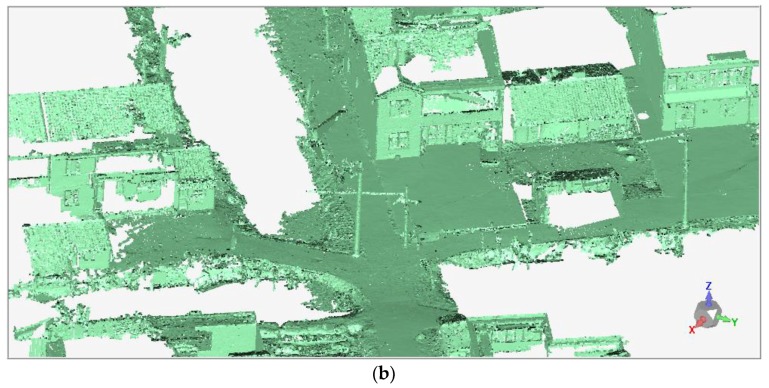
The comparison of point clouds (**a**) pre- and (**b**) post-removal of noise points.

During the laser scanning, the point clouds of unwanted objects, such as vegetation and electric wires, were inevitably acquired and were treated as noise points affecting the analysis of deformation monitoring. Therefore, both automated filtering and manual editing were adopted to identify and reduce these noise points. The automated filtering was performed based on the point density and undesired data (measurement errors, trees, and electric wires) were always characterized by sparse outliers. A point is considered as noise and removed if its average distance to neighbors is larger than a specified threshold, which can be determined according to mean and standard deviation of the nearest neighbor distance [23]. In this study, the automated filtering was performed in the Geomagic Control software. The trees and electric wires were removed through the procedure of noise reduction, while the main features of the ground surface and buildings were still maintained (Figure 4).

The whole scanning area is large, and was subdivided into 12 sections where scans were conducted respectively to collect point clouds. The point clouds of each scan are characterized by a different amount of points and need to be merged. Using white balls located in the overlapping regions, the multiple point clouds were combined into a single point cloud.

## 4. Results

### 4.1. Local Displacement Analysis

**Figure 5:**
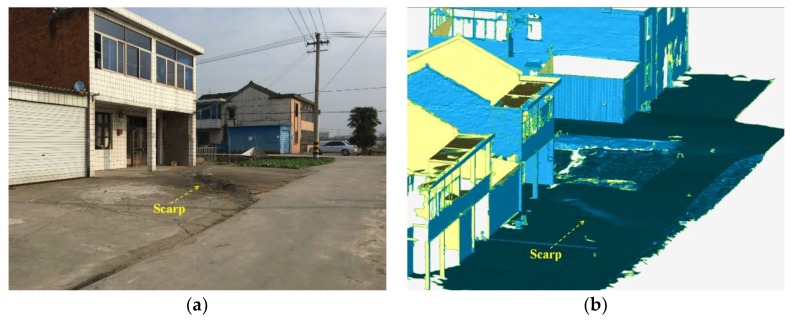

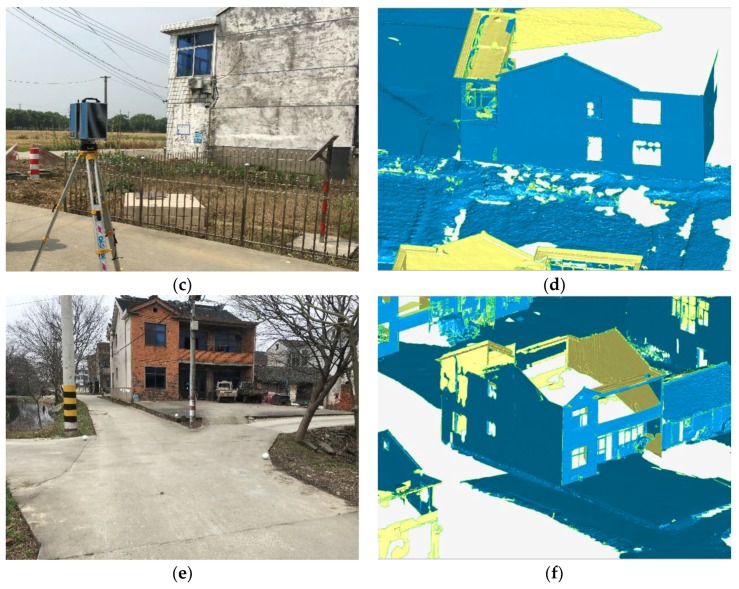
The comparison of monitoring objects in site photos and reconstructed modeling: (**a,b**) scarp, (**c**,**d**) wall A, and (**e**,**f**) wall B.

A scarp and two walls of buildings were selected for local displacement analysis. The scarp, about 6.85 m long, was discovered on a street with a strike of 11.41°. Both can be obviously observed in the site photo and the polygon model, created by triangulation. Additionally, two walls, labeled as wall A (WA) and wall B (WB), are both chosen from the two-floor houses which were distributed along the same street, consisting of 64554 and 54103 points and covering 96.85 m^2^ and 84.13 m^2^ area, respectively (Figure 5).

**Figure 6:**
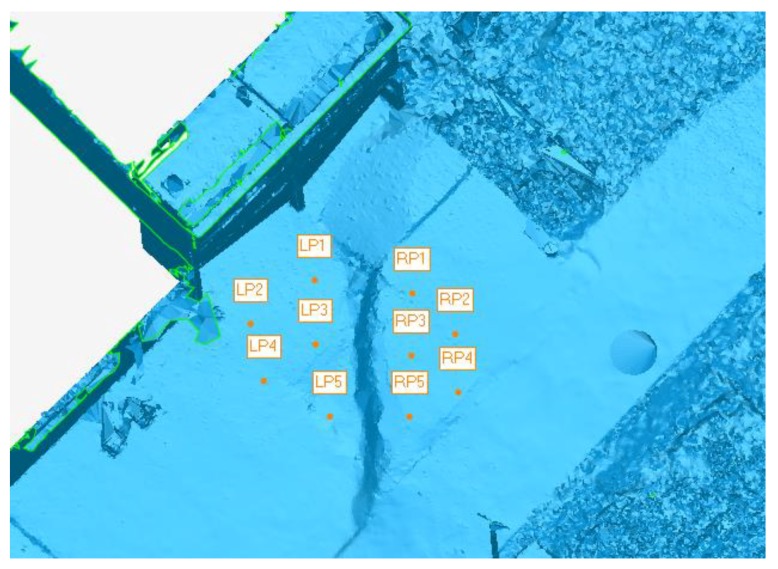
The locations of ten monitoring points installed on the left side (denoted as LP) and right side (denoted as RP) of the scarp.

**Figure 7:**
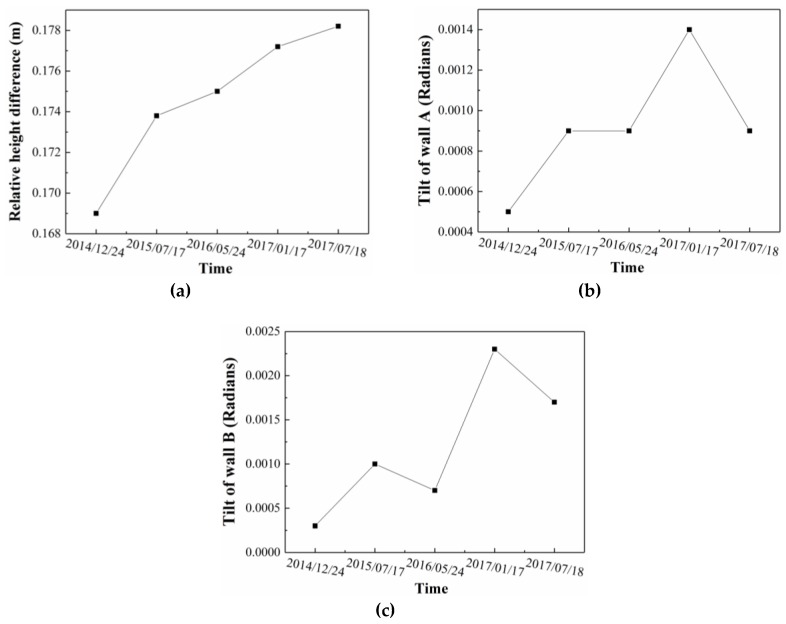
The variance of (**a**) relative height difference, (**b**) tilt of WA, and (**c**) tilt of WB with the monitoring time.

The vertical differences between the two sides of the scarp were monitored to investigate the activity of the earth fissure. In the polygon model, several monitoring points were installed on both sides of the scarp (Figure 6), and the average difference on Z-coordinate was calculated to represent the vertical difference. Figure 7a shows the variation of vertical difference with time for all five of the point clouds. Vertical difference gradually increased from 16.9 cm to 17.82 cm during the 3 years. The tilt of WA and WB were monitored to estimate the influence of the earth fissure on the surrounding buildings. The tilt of wall is defined as the tangent value of the angle between the wall and the vertical plane, which can be calculated through plane fitting to points located on the wall. The angular difference between the fitting plane and the vertical plane was computed to represent the tilt of wall. Figure 7b,c illustrates the variation of tilt with time for wall A and B, respectively. Both of them have a similar trend and the tilt of wall increases gradually as a whole. However, the magnitude of tilt is fairly close to zero, indicating the walls are kept upright with no obvious deformation.

### 4.2. Global Displacement Analysis

Global displacement analysis was performed to investigate the macroscopical deformation features by using point cloud registration. The point cloud, initially produced on 24 December 2014, was specified as the reference, target, or fixed-point cloud and the 4 remaining point clouds were assigned as test, source, or moving point clouds. Generally, in the process of rigid registration, the test point clouds can be well-aligned to reference point clouds with a minimum distance difference using the best-fit method, such as the ICP algorithm. However, because of the complexity and inhomogeneity of earth fissure hazard evolution, it is fairly difficult to capture reasonable global registration results to represent the feature of deformation-failure through the best-fit method. Therefore, in this case, a control point alignment method was adopted to address this issue [24,25,26,27]. It is necessary to select at least 3 corresponding base points on both the reference and the test surfaces to align, then the difference between the two point clouds is used to indicate the displacement distribution. Note that choosing the base points has a significant impact on the registration results and the base points are suggested to be captured in no-deformation regions, which are located on the lower right corner of point cloud.

**Figure 8:**
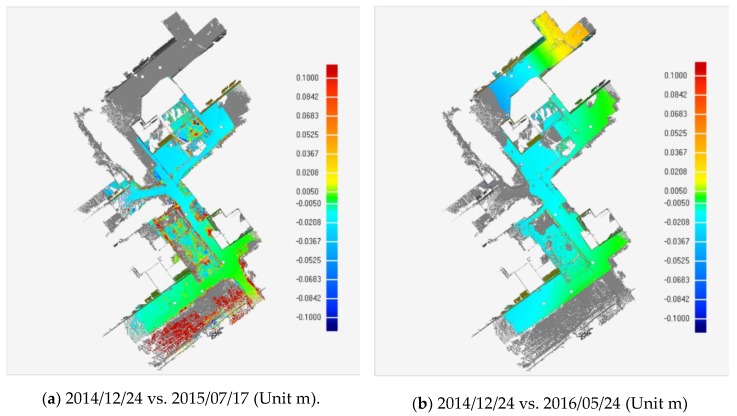

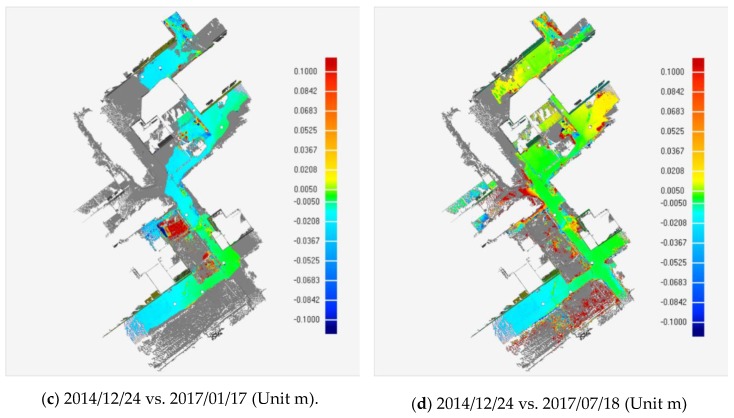
Vertical displacement distribution obtained from comparison of reference and test point clouds.

**Figure 9:**
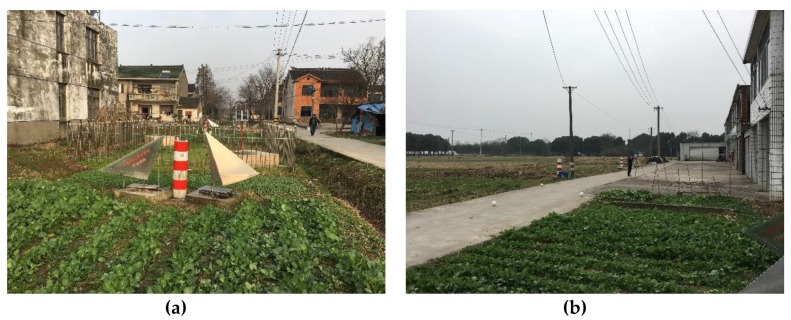
The photos of abnormal deformation regions, (**a**) monitoring facilities and (**b**) farmland.

Figure 8a–d shows the variance of vertical displacement distributions of the study area, under different monitoring times. The warm tone indicates that the ground in the southeastern part of the study area uplifts relatively and the ground surfaces in northwestern area, drawn with cool tone, move downwards relatively. The scarp on the road (Figure 5a,b) is situated just at the intersection of warm and cool tones. Furthermore, the settlement range and magnitude gradually increase with monitoring time. Noteworthily, there are some abnormal regions of deformation with crimson color in Figure 8a,c,d, indicating more ground uplifting. Based on the field survey, it was found that the large deformation areas can be mainly attributed to instrument installations and agricultural activities (Figure 9a,b).

**Figure 10:**
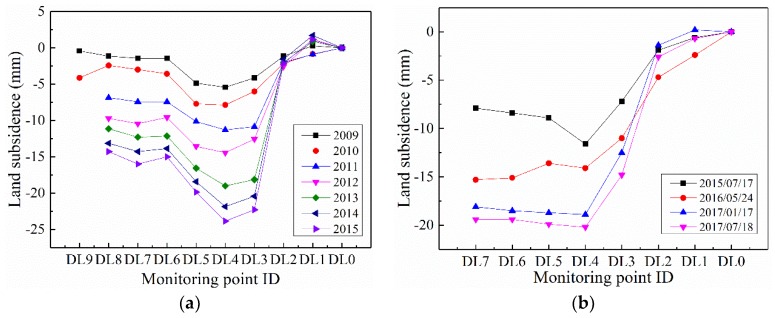
The variance of subsidence on the survey line profile with different monitoring times, based on (**a**) GPS and (**b**) TLS methods.

**Figure 11:**
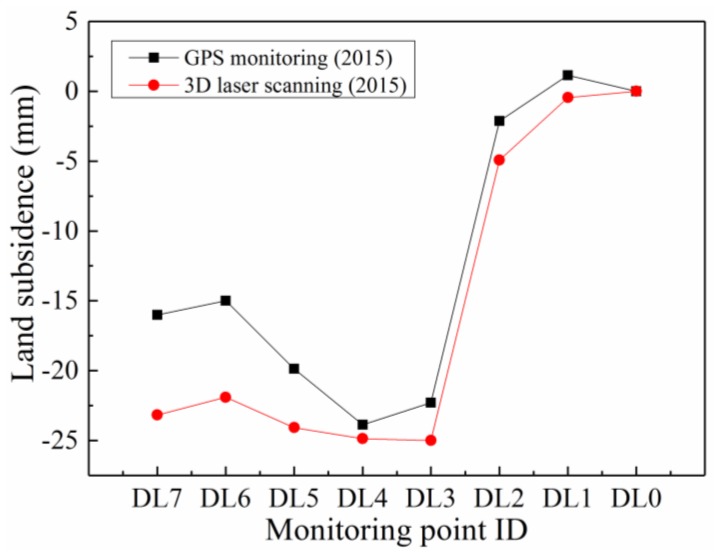
Comparison of land subsidence on the survey line in 2015, between GPS and TLS monitoring.

To validate the effectiveness and applicability of the TLS approach on earth fissure hazard monitoring, several previously installed GPS points were used to investigate the deformation features of the ground surface in the same places. Figure 10a shows the variance of ground surface settlement on the survey line, from 2009 to 2015. Obviously, larger settlements were observed at the monitoring points DL3, DL4, and DL5, which are located on the perimeter of scarp. Figure 10b illustrates the measurement results of the corresponding monitoring points using TLS. As a consequence of the influence from human activities (e.g., building construction, agricultural work), the point clouds at DL8 and DL9 are not available during the whole TLS measurement. It can be seen that similar deformation trends were obtained, and a sharp drop in vertical displacement occurred, around monitoring point DL4. Remarkably, the GPS results provided the accumulative land subsidence from 2009 annually. However, the settlement was accumulated from the first laser scanning, on 24 December 2014, in TLS monitoring. As a consequence of the presence of different monitoring periods and an accumulative start point in the two monitoring methods, it is difficult to compare the TLS data with the GPS data accurately. Therefore, it is necessary to convert TLS data into accumulative land subsidence from 2009 and to derive the annual land subsidence. More details related to the transformation will be provided in the following Discussions subsection. Finally, comparisons between the two methods were conducted at an overlapping time (2015), as shown in Figure 11. The TLS monitoring results are in acceptable agreement with GPS in vertical displacement.

## 5. Discussions

In fact, the monitoring time between TLS and GPS methods is not a one-to-one correspondence. The GPS monitoring started in 2009 to investigate the land subsidence and was terminated in 2015 for its inapplicability to areas which were influenced by human-intense activities. Alternatively, a remote sensing, TLS monitoring, was employed in the study area since 24 December 2014. Therefore, only monitoring data from 2015 can be used to make direct comparisons between the two methods. Additionally, the accumulative land subsidence was measured by years in the GPS method, which is different from that of the TLS method. To compare land subsidence between GPS and TLS monitoring in 2015, the TLS monitoring data must be transformed into the same pattern as the GPS. As aforementioned, during the global displacement analysis, the point cloud, first collected on 24 December 2014, was treated as the reference object and the other four point clouds were specified as test objects. Once the feature-based alignment was performed, the vertical displacement could be determined by moving the test point cloud to match the position of the reference point cloud and calculating the geometric differences between them. That is to say, the land subsidence in the period of 24 December 2014–17 July 2015 was obtained through comparing the first two point clouds, rather than the whole year of 2015. In this study, we assume that the average daily or monthly settlement is the same. The accumulative land subsidence in 2015 can be calculated according to the following equation:

(1) $$S_{T}=S_{b}+\frac{PC_{t}-PC_{r}}{T_{t}-T_{r}}\times T_{y}$$

where *S_T_* is the accumulative land subsidence based on TLS in 2015, *S_b_* is the accumulative land subsidence based on GPS in 2014, (*PC_t_*−*PC_r_*) represent the vertical displacement difference between the reference point cloud collected on 24 December 2014 and the test point cloud collected on 17 July 2015, respectively, (*T_t_* − *T_r_*) is the time difference between 24 December 2014 and 17 July 2015, which can be measured in days or months, and *T_y_* is the total days or months in 2015 with the same unit as (*T_t_* − *T_r_*).

More importantly, the good agreement between TLS and GPS can only be supported by monitoring points DL0–DL5. However, larger errors on monitoring points DL6 and DL7 were observed with a maximum difference of about 7 mm. Hereon, the potential reasons for the larger difference in monitoring points DL6 and DL7 between TLS and GPS approaches are listed as follows:

(1) The influences of human activities on the GPS monitoring, which is regarded as one of the most probable causes. With the development of society and the increase in population, the demand for land is on the rise. Some monitoring points (i.e., DL8 and DL9) and datum points were destroyed, which it is unavoidable in areas with intensive human activities. As DL6 and DL7 are closer to the new buildings, they will encounter more interference from human activities with higher probability.
(2) The errors introduced by data transformation are another most likely reason, as the hypothesis of the same average daily or monthly settlement is not 100% reasonable. Unfortunately, there is not enough available data to make the comparison and justify the TLS method. Therefore, the assumption has to be made to complete the data transformation.
(3) The errors coming from the process of point cloud alignment. When performing the control point alignment (e.g., feature-based alignment), at least 3 base points are required to be carefully selected, at the same positions, in both the reference and the test point clouds. Due to measurement errors and noise, some position differences may be generated, but this bias can be greatly reduced by careful calibration.

## 6. Conclusions

A high-resolution TLS monitoring approach was applied for deformation detection of the earth fissure hazard in Yangshuli Village, Wuxi City, Jiangsu Province, China. Five sets of high-density point clouds, containing millions and tens of millions of points, were collected by TLS in the interested regions from 2014 to 2017. Both local and global displacement analyses were extracted from the point clouds to capture the deformation features of the earth fissures from a small- and large-scale, respectively. From the viewpoint of local displacement, tiny deformation was observed in the position of the appointed scarp and the walls during the monitoring period. The maximum increment on vertical displacement between two sides of scarp is 1.65 cm, while no obvious inclining happened on the two walls based on tilt measurement, i.e., the tilt of the walls was approximately equal to zero. Meanwhile, global displacement analysis demonstrated that overall settlements on the ground surface increase slowly, but regions located on the left side of scarp are characterized by relatively larger vertical displacement than that in right regions. Furthermore, the TLS monitoring results match the GPS data and the actual situation in the same measuring line, demonstrating the effectiveness and applicability of the TLS technique on deformation monitoring of earth fissure hazards.

## Figures and Tables

**Table 1:** Summary of five scans.

Date	Points Count	Data Volume	Scanning Distance	Average Resolution	Scanning Area
2014/12/24	30570695	2.04 GB	<50 m	1.80 cm	56166.0984 m^2^
2015/07/17	4502430	142 MB	<50 m	2.97 cm	4843.8722 m^2^
2016/05/24	21036083	667 MB	<50 m	1.26 cm	3938.9993 m^2^
2017/01/17	14199141	444 MB	<50 m	1.85 cm	4855.5652 m^2^
2017/07/18	14940684	1.02 GB	<50 m	3.70 cm	46682.9980 m^2^
